# Rosuvastatin as a Supplemental Treatment for the Clinical Symptoms of Nephropathia Epidemica: A Pilot Clinical Study

**DOI:** 10.3390/v16020306

**Published:** 2024-02-17

**Authors:** Venera Shakirova, Maria Markelova, Yuriy Davidyuk, Robert J. Stott-Marshall, Toshana L. Foster, Svetlana Khaiboullina, Albert Rizvanov, Ekaterina Martynova

**Affiliations:** 1 Department of Infection Diseases, Kazan State Medical Academy, Kazan 420012, Russia; vene-shakirova@yandex.ru; 2 Institute of Fundamental Medicine and Biology, Kazan Federal University, Kazan 420008, Russia; mimarkelova@gmail.com (M.M.); davi.djuk@mail.ru (Y.D.); sv.khaiboullina@gmail.com (S.K.); rizvanov@gmail.com (A.R.); 3 Faculty of Medicine and Health Sciences, School of Veterinary Medicine and Science, The University of Nottingham, Sutton Bonington Campus, Loughborough LE12 5RD, UK; robert.stott-marshall@nottingham.ac.uk (R.J.S.-M.); toshana.foster@nottingham.ac.uk (T.L.F.)

**Keywords:** nephropathia epidemica, statins, rosuvastatin

## Abstract

Nephropathis epidemica (NE), a mild form of hemorrhagic fever with renal syndrome (HFRS), is an acute zoonotic disease endemic in the Republic of Tatarstan. This study aimed to assess the impact of rosuvastatin on the clinical and laboratory results of NE. A total of 61 NE patients and 30 controls were included in this study; 22 NE patients and 7 controls received a daily dose of rosuvastatin (10 mg) for ten consecutive days. Serum samples were collected on days 1, 5, and 10 after admission to the hospital. These samples were analyzed to determine the levels of lipids, cytokines, and kidney toxicity markers. Our findings indicate that rosuvastatin reduced the duration of the second wave of fever and alleviated back pain and headache symptoms. Additionally, low-density lipoprotein cholesterol (LDL-C) serum levels were significantly decreased on days 5 and 10 upon rosuvastatin treatment. Furthermore, rosuvastatin decreased the levels of cytokines in the serum, particularly proinflammatory cytokines IL-1β and IL-8. NE patients had significantly altered levels of the kidney toxicity markers albumin and osteopontin. The data from our study provide evidence supporting the therapeutic potential of rosuvastatin in NE cases.

## 1. Introduction

Nephropathia epidemica (NE) is a mild form of hemorrhagic fever with renal syndrome (HFRS); a zoonotic disease endemic in Tatarstan, Russia [1]. The infection is acquired through inhaling virus-contaminated aerosols or direct contact with infected small mammals [2]. *Orthohantavirus puumalaense* (PUUV), a member of the *Orthohantavirus* genus, is commonly isolated from NE patients and local rodents [3]. Previous studies have demonstrated that PUUV is non-cytopathic in vitro [4], suggesting indirect mechanisms of disease pathogenesis. It has been proposed that a “cytokine storm” contributes to the pathogenesis of NE, as demonstrated by the activation of chemokines and proinflammatory cytokines [5]. Additionally, changes in serum lipid composition, specifically lower high-density lipoprotein cholesterol (HDL-C) levels have been observed in NE patients [6]. Our research has also revealed an association between low triglyceride levels and the upregulation of interferon γ (IFNγ) and interleukin (IL)-12 in NE serum [6]. Therefore, it could be suggested that the decreased level of HDL-C and cytokine-induced inflammation are contributing factors in the pathogenesis of NE.

Orthohantaviruses are enveloped viruses that use membrane fusion mechanisms to release ribonucleocapsids into the cytoplasm [7]. The composition of membrane lipids therefore contributes to the early stages of virus entry. Cifuentes-Munoz et al., demonstrated that cholesterol depletion of cell membranes significantly reduced orthohantavirus infectivity [8]. Another study, by Chiang et al., also identified cholesterol-dependent mechanisms as one of the main pathways for orthohantavirus entry [9]. Cholesterol is a component of raft domains [10], which play a role in sorting membrane molecules, receptor expression, and signal transduction [11]. The available evidence suggests that cell membrane cholesterol contributes to orthohantavirus infection, as depletion of this molecule leads to decreased infectivity of orthohantaviruses [12,13]. It has been proposed that the interaction between orthohantavirus glycoproteins and the cell membrane depends on the abundance of cholesterol.

NE is characterized by activating inflammatory mediators and cytokines [14,15,16]. Previous studies have demonstrated that serum levels of tumor necrosis factor α (TNF-α) and IL-1β correlate with the severity of the disease [14,17]. Additionally, plasma levels of IL-6 are a suggested indicator of NE severity [16]. Serum levels of IL-6, as well as the chemokines CXCL10, CCL2, and CCL3, have been found to correlate with the clinical symptoms of NE [14]. Our data also identified multiple upregulated cytokines in the serum of NE patients, further supporting the role of a “cytokine storm” in the pathogenesis of the disease [15]. In addition to contributing to disease severity and symptoms, increased serum cytokine levels may also affect lipid composition as TNFα, IL-1, and IFNα can stimulate hepatocyte fatty acid synthesis in vitro [18]. This increased lipid synthesis was observed when doses of cytokines used were similar to those that induce fever in vivo [19], a common physiological reaction to elevated cytokine levels [20]. Notably, triglycerides can further promote TNF-α production by leukocytes, establishing a positive feedback loop [21].

Orthohantavirus sensitivity to cholesterol levels suggests that disruption of sterol synthesis may be a potential target for therapeutic development [12]. Statins are a group of drugs that reduce cholesterol biosynthesis [22] by inhibiting the 3-hydroxy-3-methyl-glutaryl coenzyme A (HMG-CoA) reductase, a rate-limiting enzyme in cholesterol synthesis [23] and could be a supplemental treatment option for NE and HFRS. We therefore aimed to investigate the therapeutic efficacy of rosuvastatin, a statin that inhibits HMG-CoA reductase [24]. Additionally, rosuvastatin targets hepatocytes to increase the expression of low-density lipoprotein cholesterol (LDL-C) receptors [25], which could potentially lower LDL-C levels in the serum by increasing lipid uptake from circulation.

We analyzed the therapeutic efficacy of rosuvastatin (10 mg/day for 10 days) on clinical symptoms and laboratory data in patients with NE. We then evaluated the effects of the statin on NE symptoms, lipid and cytokine levels in the serum, and markers of kidney toxicity.

## 2. Materials and Methods

### 2.1. Subjects

Serum samples were collected from 61 patients diagnosed with moderate NE and 30 controls at the Agafonov Republican Clinical Hospital for Infectious Diseases in the Republic of Tatarstan. The serum samples were collected on days 1, 5, and 10 after hospitalization. Additionally, urine samples were collected on day 10 of hospitalization. Clinical and laboratory records of the patients were also collected. The diagnosis of NE was established based on clinical presentation and confirmed serologically by detecting anti-orthohantavirus antibodies using ELISA. The samples were collected following the standard operating procedure protocol used in the hospital for diagnosing orthohantavirus infection and stored at −80 °C until further use.

### 2.2. The Severity of the Disease

The severity of the disease was determined according to the National Diagnostic Criteria for infectious diseases by Yuschuk and Vengerov, as well as recently updated criteria [26]. Moderate NE was characterized by fever (39.5 °C), headache, frequent vomiting, lumbar pain, abdominal pain, hemorrhages, oliguria (<300 mL/day), increased serum levels of blood urea nitrogen (BUN) (>18 mM/L), and serum creatinine (sCr) (>300 µM/L).

### 2.3. Statin Treatment

All 61 patients received standard therapy for NE, which aimed to maintain fluid and electrolyte balance and control fluid output. None of these patients developed severe thrombocytopenia requiring platelet transfusion, and hemodialysis was not required. A sub-group of 22 patients received rosuvastatin (ros-NE; 10 mg once daily for ten days) in addition to standard therapy. Blood and serum samples were collected from these patients on day one of hospitalization, prior to the initiation of treatment. Follow-up samples were collected on days 5 and 10. The patients receiving only standard therapy and those receiving standard therapy combined with rosuvastatin were similar in age, sex, and disease severity.

### 2.4. Controls

Of the controls, 7 received rosuvastatin (ros-control; 10 mg once per day for ten days). Serum samples were collected from these controls on day one before receiving the first dose of rosuvastatin and on days 5 and 10 during the treatment period.

### 2.5. Inclusion Criteria

Males and females aged 17–90 years old diagnosed with a moderate form of NE were included in this study.

### 2.6. Exclusion Criteria

Patients with mild and severe forms of NE were excluded. Patients younger than 17 years old were also omitted. Additionally, patients with co-morbidities including diabetes, hypertension, chronic kidney insufficiency, and cancer were excluded from this study.

### 2.7. Ethics Statement

The Ethics Committee of the Kazan State Medical Academy (KSMA) approved this study, and signed informed consent was obtained from each patient and control according to the guidelines adopted under this protocol (protocol 6/11 of the meeting of the Ethics Committee of the KSMA dated 26 November 2020).

### 2.8. Othohantavirus ELISA

The Hantagnost diagnostic ELISA kit (Institute of Poliomyelitis and Viral Encephalitis, Moscow, Russia) was used to detect hantavirus-specific antibodies following the manufacturer’s instructions. Briefly, serum samples from NE patients and controls were diluted 1:100 in PBS and incubated for 60 min at 37 °C in a 96-well plate coated with pre-adsorbed orthohantavirus antigens. After three washes (0.5% Tween20 in PBS, PBS-T), the wells were incubated with anti-human IgG-HRP conjugated antibodies (1:10,000 in PBS-T, American Qualex Technologies, San Clemente USA) for 30 min at 37 °C. Subsequently, the wells were washed three times with 0.5% Tween20 in PBS, followed by incubation with 3,3′,5,5′ Tetramethylbenzidine (Chema Medica, Moscow, Russia). The reaction was stopped by adding 10% phosphoric acid (TatKhimProduct, Kazan, Russia). The data were measured using a microplate reader Tecan 200 (Tecan, Switzerland) at OD450 with a reference OD650.

ELISA results were calculated using following equation:

PC = ODs/ODc

PC—positivity coefficient;

ODs—optic density at 450 of the patient’s serum sample;

ODc—optic density critical.

ODc = ODcontr + 0.3

ODcont—optic density of negative control serum samples.

### 2.9. Serum Lipid Analysis

Cholesterol levels were determined using a Cholesterol LiquiColor Test on the Humastar 600 biochemical analyzer as per the manufacturer’s instructions. A measurement method of an enzymatic colorimetric (CHOD-PAP) assay with an anti-lipid factor was used.

The level of triglycerides was determined using a Triglycerides LiquiColor Test (Mono) on the Humastar 600 biochemical analyzer, according to the manufacturer’s instructions. A measurement method of an enzymatic colorimetric (GPO-PAP) assay with an anti-lipid factor was used.

The level of LDL-C and HDL-C was determined using an LDL Cholesterol LiquiColor Test or HDL Cholesterol LiquiColor Test, respectively on the Humastar 600 biochemical analyzer as per the manufacturer’s instructions with a direct homogeneous enzymatic measurement method.

### 2.10. RT-PCR Detection and Sequencing of PUUV Transcripts

Total RNA was extracted from 100 μL of blood using the TRIzol^®^ reagent (Life Technologies, Carlsbad, CA, USA). cDNA synthesis was performed using the Thermo Scientific RevertAid Reverse Transcriptase (Thermo Fisher Scientific, Waltham, MA, USA) following the manufacturer’s instructions. Two rounds of PCR were conducted to amplify the S segment target sequences. The PCR products were subsequently sequenced to confirm the orthohantavirus strain. The primers used for the first round of amplification were: PUUV-39S-F3 (forward) and PUUV-S-R1496 (reverse) [27]. For the second round of amplification, the primers used were PUUV-S-F704 (forward) [27] and PUUV-S-R1496 (reverse), resulting in a product of 836 bp.

PCR products were purified using the Isolate II PCR and Gel Kit () and subjected to sequencing using the ABI PRISM 3730 Big Dye Terminator 3.1 sequencing kit (ABI, Waltham, MA, USA). The obtained sequences were deposited in the GenBank database under the accession no. OR420714-OR420724.

Phylogenetic analysis of PUUV sequences was conducted using the maximum likelihood method based on the Tamura–Nei model in the MEGA v6.0 software [28]. The analysis included several S-segment sequences of the GenBank PUUV strains recently isolated from bank voles in the RT and some other regions of Russia. The sequence of the *Tula orthohantavirus* S segment was used as an outgroup.

### 2.11. Multiplex Analysis

A total of 48 analytes were analyzed in serum samples using Bio-Plex multiplex magnetic bead-based antibody detection kits (Bio-Rad, Hercules, CA, USA), following the manufacturer’s instructions. In this study, we used the Bio-Plex Pro Human Cytokine 21-plex and Bio-Plex Human Cytokine 27-plex panels. Urine samples were analyzed using the Bio-Rad Human Kidney Toxicity Panel 2 (Bio-Rad, Hercules, CA, USA), which detects albumin, beta-2-microglobulin (β2M), cystatin C, neutrophil gelatinase-associated lipocalin (NGAL), osteopontin, and trefoil factor 3 (TFF3). Serum or urine aliquots of 50 μL were analyzed, and a minimum of 50 beads per analyte were acquired. Each analysis included standards and quality controls. Median fluorescence intensities were measured using a Luminex 100 or 200 analyzer (Luminex, Austin, TX, USA). Each sample was analyzed in triplicate. Standard curves for each cytokine were generated using standards provided by the manufacturer, and data analysis was performed using the MasterPlex CT control software 1.0 and MasterPlex QT analysis software (MiraiBio, Alameda, CA, USA).

### 2.12. Statistical Analysis

Statistical analysis was conducted using the R environment 2.0 [29]. A *p*-value < 0.05 was considered statistically significant and was determined using the Kruskal–Wallis test with Benjamini–Hochberg adjustment for multiple comparisons.

## 3. Results

### 3.1. PUUV Genetic Variations in NE Patients

To confirm that variations in clinical signs and symptoms were not due to differences in infecting strains of PUUV, blood samples were collected on day 1 of hospitalization and used for RNA extraction to analyze PUUV genetic variants. Orthohantavirus RNA was detected in 14 and 9 NE and ros-NE patients, respectively. The partial S segment sequences, 633 nt long, of the PUUV strains were isolated from five NE and six ros-NE patients. We found that all identified PUUV strains are closely related to the strains circulating in the bank vole populations in the RT belonging to the Russian (RUS) genetic lineage [27,30,31] (Supplemental Figure S1). We also found that PUUV strains isolated from each group of patients did not form a separate clade and were grouped.

### 3.2. Patients

All 61 patients received standard treatment upon admission to the hospital. NE patients were diagnosed with a moderate form of the disease, which did not necessitate thrombocyte transfusion or hemodialysis. Rosuvastatin therapy was initiated on day 1 and continued for ten days. Blood samples were collected on three occasions during hospitalization: on days 1, 5, and 10. The clinical signs and symptoms of the NE and ros-NE patients are summarized in Table 1.

### 3.3. Analysis of Clinical Laboratory Data in NE Patients and Controls

In NE patients without rosuvastatin, there were increased serum levels of urea on days 1 and 5, while a prolonged increase in creatinine, alanine aminotransferase (ALT), and aspartate aminotransferase (AST) levels was observed on days 1, 5, and 10 compared to controls (Table 2). Low serum levels of HDL-C were detected on days 1, 5, and 10 compared to the controls (Table 3).

### 3.4. Analysis of Rosuvastatin Treatment on Clinical Signs and Symptoms in NE Patients

Rosuvastatin reduced the duration of the second wave of fever, lumbar pain, and headache compared to that in NE without rosuvastatin treatment (Table 1). Additionally, ros-NE patients had higher serum levels of anti-orthohantavirus IgM and IgG antibodies compared to NE patients (Table 2).

### 3.5. The Effect of Rosuvastatin on Serum Lipid Levels

The impact of rosuvastatin on serum lipid levels in ros-NE patients and the corresponding controls was also assessed. In the controls, rosuvastatin treatment had no significant effect on serum levels of HDL-C, LDL-C or triglycerides (Table 3).

Serum levels of triglycerides were higher in the NE patients than in controls. This increase was not inhibited by rosuvastatin treatment and serum triglyceride levels remained significantly higher than corresponding controls at all time points (Table 3). NE patients had significantly lower serum HDL-C than the controls; however, rosuvastatin treatment did not affect these levels. Furthermore, serum LDL-C was reduced in NE patients compared to controls although not statistically significantly. These LDL-C levels were further reduced by rosuvastatin treatment in NE patients and remained lower than in control patients with or without rosuvastatin treatment (Table 3).

### 3.6. Effect of Rosuvastatin on Urine Kidney Toxicity Markers in NE Patients

**Figure 1:**
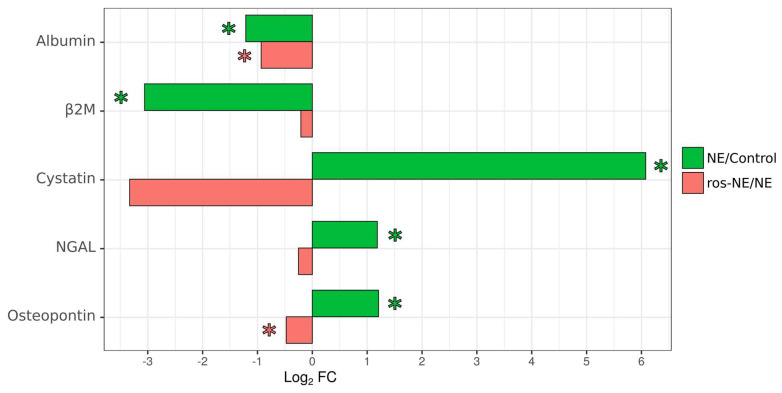
Urine levels of kidney toxicity markers in NE patients. Kidney toxicity markers in the urine were analyzed using Bio-Rad Human Kidney Toxicity Panel 2. Data are presented as a log2-fold change in NE patients relative to controls (green) or ros-treated NE patients relative to untreated NE patients (red). Asterisks denote statistical significance (*p* < 0.05) determined by Kruskal–Wallis test with Benjamini–Hochberg adjustment for multiple comparisons.

The impact of rosuvastatin on kidney function was assessed using the Human Kidney Toxicity Panel 2 Immunoassay (Bio-Rad, Hercules, CA, USA). There were limited effects of rosuvastatin on the levels of kidney toxicity markers in the controls (Figure 1; Supplemental Table S1). In NE patients without treatment, lower levels of albumin and β2M were observed, while cystatin, NGAL, and osteopontin were increased compared to the corresponding controls (Figure 1; Supplemental Table S1). In ros-NE patients, lower levels of albumin and β2M and higher levels of NGAL were found compared to the corresponding controls (Supplemental Table S1). Interestingly, lower levels of albumin and osteopontin were found in ros-NE compared to NE patients.

### 3.7. Effect of Rosuvastatin on Serum Cytokines in NE Patients

**Figure 2:**
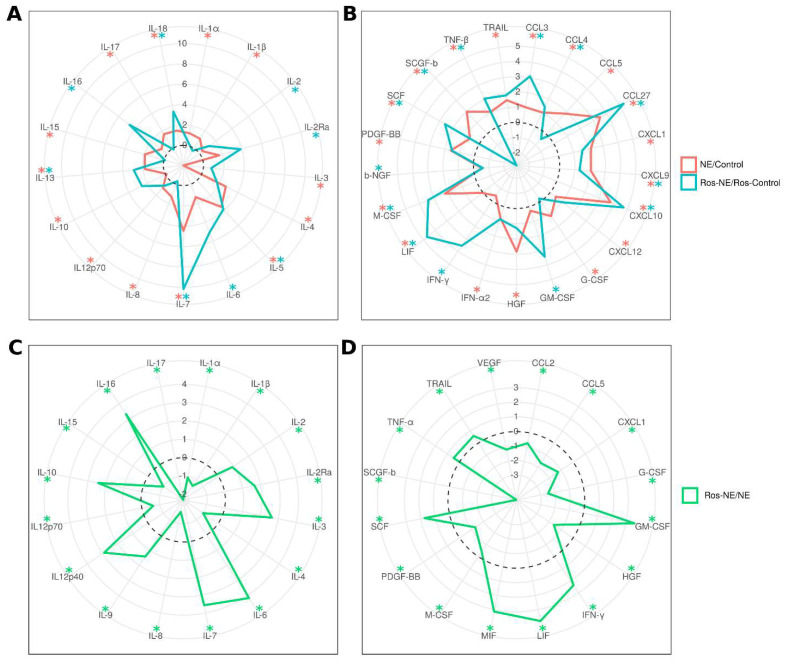
Effect of rosuvastatin on serum cytokine levels in NE patients. Serum cytokine levels were analyzed using the Bio-Plex Pro Human Cytokine 21-plex and Bio-Plex Human Cytokine 27-plex panels. Levels of interleukins (**A**) or cytokines, chemokines, and growth factors (**B**) in NE patients relative to controls (red) and in ros-treated NE patients relative to ros-treated controls (blue). Levels of interleukins (**C**) and cytokines, chemokines, and growth factors (**D**) were also measured in ros-treated NE patients relative to untreated NE patients (green). Data are presented as a log2−fold change. Asterisks denote statistical significance determined by Kruskal–Wallis test with Benjamini–Hochberg adjustment for multiple comparisons.

Inflammatory cytokine levels are often found elevated in the NE patients’ serum [14,16,28], suggesting their potential role in the pathogenesis of the disease. Therefore, our study aimed to investigate the impact of rosuvastatin on serum cytokine levels (Figure 2).

We observed increased levels of 28 cytokines (IL-1α, IL-1β, IL-4, IL-5, IL-7, IL-8, IL12p70, IL-13, IL-15, IL-17, IL-18, CCL3, CCL4, CCL5, CCL27, CXCL1, CXCL9, CXCL10, CXCL12, G-CSF, HGF, IFN-α2, LIF, M-CSF, PDGF-BB, SCF, SCGF-β, TNF-β, and TRAIL) in the serum of NE patients compared to the controls without statin treatment (Figure 2, red line and asterisks). Only two cytokines, IL-3 and IL-10, were lower in NE patients than in the controls.

Levels of only 19 cytokines (IL-2, IL-2Ra, IL-5, IL-6, IL-7, IL-13, IL-16, IL-18, CCL3, CCL4, CCL27, CXCL9, CXCL10, GM-CSF, IFN-γ, LIF, M-CSF, SCF, and TNF-β) were higher in ros-NE patients compared to the rosuvastatin-treated controls (Figure 2, blue lines and asterisks). The levels of proinflammatory cytokines IL-1α, IL-1β, and IL-8 found elevated in NE patients, were not affected in ros-NE patients compared to corresponding controls. Also, two cytokines (β-NGF and SCGF-β) were lower in ros-NE patients than in the rosuvastatin-treated controls (Figure 2, blue line and asterisks).

Next, we compared serum cytokine levels in NE and ros-NE patients (Figure 2). We observed lower levels of 16 cytokines (IL-1α, IL-1β, IL-4, IL-8, IL12p70, IL-15, IL-17, CCL2, CCL5, CXCL1, G-CSF, HGF, M-CSF, PDGF-BB, SCGF-β, and VEGF) in ros-NE compared to NE patients. Additionally, we identified a subset of 16 cytokines (IL-2, IL-2Ra, IL-3, IL-6, IL-7, IL-9, IL12p40, IL-10, IL-16, GM-CSF, IFN-γ, LIF, MIF, SCF, TNF-α and TRAIL) that were higher in ros-NE compared to NE patients (Figure 2).

## 4. Discussion

We found that rosuvastatin had a beneficial effect on the severity of specific clinical symptoms in NE cases. A single dose of rosuvastatin administered for ten days reduced the duration of the second wave of fever, lumbar pain, and headache. These findings suggest that including rosuvastatin as a supplementary treatment could improve NE symptoms. We believe that the effect of rosuvastatin was linked to its anti-inflammatory effects which have been demonstrated in vitro [32] and in vivo [33,34]. It has been suggested that the inhibitory effect of statins on inflammation is due to the reduced production of proinflammatory cytokines [32,35]. In this study, rosuvastatin reduced the serum levels of only a few proinflammatory cytokines, namely IL-1α, IL-1β, and IL-8.

IL-1α and IL-1β belong to the IL-1 cytokine family, which is frequently associated with pathological inflammation [36]. The role of IL-1α in the pathogenesis of inflammation is supported by multiple studies [37,38,39]. It has been suggested that IL-1α, released by damaged or stressed cells, establishes an “inflammatory loop” by recruiting inflammatory hematopoietic cells to the site of infection [40]. These hematopoietic cells maintain the inflammatory environment by enhancing the release of inflammatory cytokines.

We observed a decreased serum level of IL-1β in ros-NE patients. IL-1β is a product of activated inflammasomes [41]. Its role in the pathogenesis of inflammation is established as a critical component of the “cytokine storm”, which can lead to tissue injury [42,43]. Reports have demonstrated the therapeutic potential of blocking IL-1β in patients with acute infections and autoimmune diseases [44,45,46]. Furthermore, our findings indicated that rosuvastatin reduced the serum level of IL-17, a proinflammatory cytokine [47]. It appears that IL-1β may prime pathogenic γδT17 and Th17 cells [48] and synergize with IL-17 to recruit neutrophils to the site of inflammation [49]. IL-8 is stimulated by IL-1β [50] and was lower in NE patients treated with rosuvastatin. IL-8 is a potent activator of neutrophils and a key mediator of autocrine and paracrine inflammation [51,52].The decreased level of IL-1β observed likely contributes to the lower levels of IL-8 measured in NE patients treated with rosuvastatin. These data support the hypothesis that the therapeutic efficacy of rosuvastatin in NE patients may be attributed to a reduced inflammatory response resulting from decreased release of proinflammatory cytokines.

Additionally, we observed that rosuvastatin decreased LDL-C serum levels on days 5 and 10 in NE patients. LDL-C molecules act as major cholesterol carriers in the blood, delivering it to tissues with high sterol demands [33]. These sites could include infected endothelial cells, as orthohantavirus infection increases the demand for cholesterol in cell membranes [13]. A study by Kleinfelter et al., demonstrated that orthohantavirus infection requires high cholesterol concentrations in cellular membranes for fusion between the viral and cell membranes [13]. The authors suggest the therapeutic potential of lowering serum cholesterol levels in patients with orthohantavirus infection. Similar suggestions were made by Petersen et al. [12]. The role of cholesterol in the entry of PUUV and DOBV orthohantaviruses causing NE has been shown by Leonovich and Dzagurova [53]. The authors demonstrated reduced infectivity of these orthohantaviruses in vitro after treatment with statins. It was suggested that the reduced infectivity resulted from lower cholesterol levels in the culture medium of cells treated with statins. Our results demonstrate that the level of LDL-C, the primary carrier of cholesterol in the serum, was reduced in patients treated with rosuvastatin. This reduction in LDL-Cs could lead to a decreased cholesterol supply to the orthohantavirus replication site.

Another contribution to the therapeutic efficacy of lowering LDL-C is their role in the pathogenesis of inflammation. LDL-C can contribute to inflammasome activation by depositing cholesterol into the endothelium [54]. This cholesterol deposition can form crystals capable of activating the inflammasome and releasing IL-1β [55]. The deposition of cholesterol is enhanced when endothelial cells are damaged and produce a large quantity of extracellular matrix proteins with a high affinity for LDL-C [55]. These high-LDL-C-affinity proteins create a positive feedback loop that promotes cholesterol deposition. As a result, a disturbed cholesterol accumulation in the endothelial wall could contribute to the hypertension observed in convalescent NE patients [56,57].

Regarding kidney injury in NE, we analyzed urine levels of albumin, β2M, cystatin C, NGAL, osteopontin, and TFF3 as markers of kidney toxicity. Our findings demonstrated signs of kidney injury in NE, supporting our previous report [58]. Interestingly, rosuvastatin substantially reduced the extent of deviation in two of these kidney toxicity markers in NE compared to controls. The markers affected in rosuvastatin-treated NE compared to untreated NE patients were albumin and osteopontin, while changes in β2M, cystatin, NGAL, and TFF3 levels were limited. Markers of tubular injury, such as cystatin and NGAL, were elevated in NE patients compared with uninfected controls, which supports the hypothesis of tubular injury/necrosis as an explanation for AKI in NE [59,60]. We found that rosuvastatin reduced the urine level of cystatin, although this was not statistically significant.

Two kidney toxicity markers were reduced in ros-NE patients compared to NE patients: albumin and osteopontin. Urine albumin levels are commonly used as a diagnostic and prognostic marker of kidney injury [61]. A study by Yu et al. has demonstrated that urinary albumin was a better indicator of kidney tubular injury compared to TFF3 [62]. It should be noted that, in NE, histological patterns of kidney tissue damage are explained as tubulointersticial nephritis with tubular proteinuria [63,64]. Osteopontin is another marker of AKI [65]. This was observed in tubular epithelial cells, suggesting that the expression of this protein could be changed during tubular damage [66,67]. Indeed, increased expression of osteopontin was demonstrated in the kidney epithelial cells of small mammal models of hypoxia-induced kidney damage [68], polycystic kidney disease [69], and angiotensin II-induced tubulointerstitial nephritis [70]. It has been suggested that osteopontin can contribute to the pathogenesis of kidney injury by attracting Th1 cells and by supporting Th1 and Th17 differentiation [71,72,73]. However, a protective role of osteopontin has also been demonstrated as it was shown to reduce apoptosis and promote regeneration and repair of tubular cells [74]. Reduced osteopontin in ros-NE urine may indicate lesser recruitment of pathologic leukocytes to the kidney tissue compared to that in NE. Additionally, lower osteopontin could indicate a limited requirement for epithelial repair, suggesting restricted tubular damage. This assumption is supported by the lower observed levels of urinary albumin in ros-NE patients compared to NE patients. Therefore, reduced urine albumin and osteopontin in ros-NE patients compared to NE patients suggest the limited tubular injury could be attributed to statin treatment.

It is worth noting that rosuvastatin had limited interference with the development of the humoral immune response in NE patients. This statement is supported by the finding that the serum levels of anti-orthohantavirus IgM and IgG were higher in NE patients with statin treatment than those without. Our data indicate that rosuvastatin may reduce the severity of NE clinical symptoms without affecting the immune response.

In conclusion, we demonstrate that rosuvastatin reduces LDL-Cs in NE patients compared to the corresponding controls (Table 3). Additionally, rosuvastatin improves kidney tubular function, as evidenced by the reduction in kidney toxicity markers in statin-treated patients compared to untreated patients (Figure 1, Supplemental Table S1). Also, there was significant reduction in some clinical symptoms such as fever, back pain, diarrhea and headache (Table 1). These effects of statins are likely attributed to their anti-inflammatory properties as serum level of pro-inflammatory cytokines IL-1α, IL-1β, and IL8 were lower in ros-NE compared to NE (Figure 2). These data provide some evidence for the therapeutic potential of rosuvastatin in NE. However, it is important to acknowledge that this study had a small group of patients, and further validation through large cohort studies and potential dosage alterations would be beneficial to confirm the therapeutic efficacy of statins in NE.

## Figures and Tables

**Table 1:** Clinical signs and symptoms of NE patients.

	NE	ros-NE	*p* Value
Sex (m/f)	36/3	21/1	1 *
Age (years)	36.23 ± 11.08	36.95 ± 12.63	0.93 ^§^
Fever 1 (days)	6.44 ± 2.73	5.64 ± 2.15	0.27 ^§^
Fever 2 (days)	0.82 ± 1.70	0.00 ± 0.00	0.01 ^§^
Lower back pain (days)	6.54 ± 4.25	4.09 ± 2.16	0.02 ^§^
Vomiting (yes/no)	12/27	7/15	1 *
Diarrhea (yes/no)	15/24	3/19	0.047 *
Headache (days)	6.21 ± 2.91	3.91 ± 1.02	0.001 ^§^
Insomnia (days)	2.58 ± 3.09	1.82 ± 1.76	0.6 ^§^

^§^—Kruskall–Wallis test. *—Exact Fisher test.

**Table 2:** Clinical laboratory test results for all patients.

	Days	NE	Control	NE vs. Control (*p* Value)	ros-NE	ros-Control	ros-NE vs. ros-Control (*p* Value)	NE vs. ros-NE (*p* Value)
Urea (mM/L)	1	10.77 ± 8.95	4.42 ± 1.07	<0.001	8.07 ± 3.70	4.17 ± 0.65	0.005	0.77
	5	11.46 ± 9.65		<0.001	8.27 ± 4.35		0.002	0.93
	10	5.27 ± 1.65		0.10	5.72 ± 2.62		0.07	0.91
Creatinine (mg/dL)	1	210.77 ± 206.13	85.22 ± 11.84	<0.001	156.09 ± 75.21	86.14 ± 10.19	0.002	0.29
	5	204.18 ± 178.03		<0.001	156.64 ± 96.35		0.002	0.95
	10	105.95 ± 34.06		<0.001	100.36 ± 21.32		0.17	0.30
ALT (units/L)	1	51.35 ± 32.27	20.04 ± 4.32	<0.001	71.03 ± 34.65	16.71 ± 4.42	<0.001	0.21
	5	58.26 ± 41.74		<0.001	68.79 ± 36.93		<0.001	0.36
	10	71.78 ± 81.72		<0.001	80.00 ± 58.50		<0.001	0.60
AST (units/L)	1	65.88 ± 58.06	22.35 ± 3.47	<0.001	72.66 ± 38.62	20.29 ± 4.50	<0.001	0.25
	5	53.51 ± 28.53		<0.001	61.30 ± 26.64		<0.001	0.28
	10	46.09 ± 28.93		<0.001	46.50 ± 25.73		<0.001	0.72
Leukocytes (cells/μL ×10^9^)	1	9.91 ± 3.45			10.96 ± 3.35			0.24
	5	10.21 ± 3.58			11.21 ± 3.34			0.16
	10	9.16 ± 2.25			9.55 ± 2.27			0.56
Platelets (cells/μL)	1	91.05 ± 54.74			91.05 ± 61.51			0.82
	5	171.00 ± 101.25			170.14 ± 76.59			0.74
	10	261.25 ± 95.92			272.09 ± 54.93			0.97
Hemoglobin (g/dL)	1	144.92 ± 29.55			154.09 ± 21.17			0.37
	5	135.56 ± 15.73			141.59 ± 14.26			0.16
	10	139.21 ± 10.67			143.86 ± 14.67			0.23
ESR (mm/h)	1	14.39 ± 9.83			14.59 ± 4.82			0.38
	5	19.41 ± 14.38			14.00 ± 5.68			0.43
	10	19.18 ± 11.74			12.77 ± 5.48			0.11
Urine protein (mg/dL)	1	0.70 ± 0.90			0.83 ± 2.07			0.85
	5	0.07 ± 0.17			0.01 ± 0.06			0.09
Urine gravity	1	1014.92 ± 7.48			1015.14 ± 8.45			0.98
	5	1009.59 ± 5.88			1008.23 ± 4.70			0.44
	10	1014.92 ± 4.94			1016.05 ± 5.28			0.50
Urine volume (ml/day)	1	883.46 ± 706.31			938.18 ± 577.58			0.57
	5	2152.44 ± 1150.92			1976.82 ± 1437.28			0.26
	10	2188.21 ± 892.52			2210.91 ± 1023.77			0.74
Potassium (mEq/L)	1	4.06 ± 0.53			4.32 ± 0.53			0.08
	5	4.16 ± 0.53			4.39 ± 0.58			0.15
	10	4.20 ± 0.36			4.28 ± 0.54			0.61
antibody (PC)	IgM IgG	11.6 ± 5.2 13.1 ± 7.4			13.8 ± 3.6 19.2 ± 8.0			0.04 0.005

ESR—erythrocytes sedimentation rate; Antibody—anti-orthohantavirus antibodies analyzed using the Hantagnost diagnostic ELISA kit (Institute of Poliomyelitis and Viral Encephalitis, Moscow, Russia); PC—positivity coefficient. Anti-orthohantavirus antibodies were analyzed in serum collected on the 5th day of hospitalization.

**Table 3:** Serum lipids in NE and ros-NE patients.

	Days	NE	Control	NE vs. Control (*p* Value)	ros-NE	ros-Control	ros-NE vs. ros-Control (*p* Value)	NE vs. ros-NE (*p* Value)	Control vs. ros-Control (*p* Value)
HDL-C (mM/L)	5	0.92 ± 0.18	2.56 ± 0.82	>0.001	0.84 ± 0.25	1.82 ± 0.65	0.001	0.40	0.47
	10	1.04 ± 0.21	2.50 ± 1.30	>0.001	1.01 ± 0.37	1.41 ± 0.64	0.11	0.34	0.12
LDL-C (mM/L)	5	2.40 ± 0.81	3.13 ± 1.05	0.14	1.64 ± 0.76	2.86 ± 1.03	0.004	0.003	0.67
	10	2.59 ± 0.88	3.26 ± 1.02	0.27	1.87 ± 1.03	2.77 ± 1.80	0.27	0.01	0.25
Triglyceride (mM/L)	5	2.64 ± 1.70	0.82 ± 0.16	>0.001	2.46 ± 0.93	0.80 ± 0.22	>0.001	0.74	0.99
	10	2.87 ± 1.60	1.15 ± 0.38	>0.001	2.55 ± 0.89	1.27 ± 0.53	0.004	0.97	0.77

## Data Availability

Data are contained within the article and supplementary materials.

## Supplementary Materials

The following supporting information can be downloaded at: https://www.mdpi.com/article/10.3390/v16020306/s1, Figure S1: Phylogenetic Tree; Table S1: Analysis of kidney toxicity markers (ng/mL) in NE.
