# Supplementary material for: Rosuvastatin as a Supplemental Treatment for the Clinical Symptoms of Nephropathia Epidemica: A Pilot Clinical Study
Source: Viruses. 2024 Feb 17;16(2):306. doi: 10.3390/v16020306 (PMC10892398; doi:10.3390/v16020306)
Supplement: Supplementary file 1 [file viruses-16-00306-s001.zip › viruses-2801373-supplementary.pdf]

Supplementary Materials

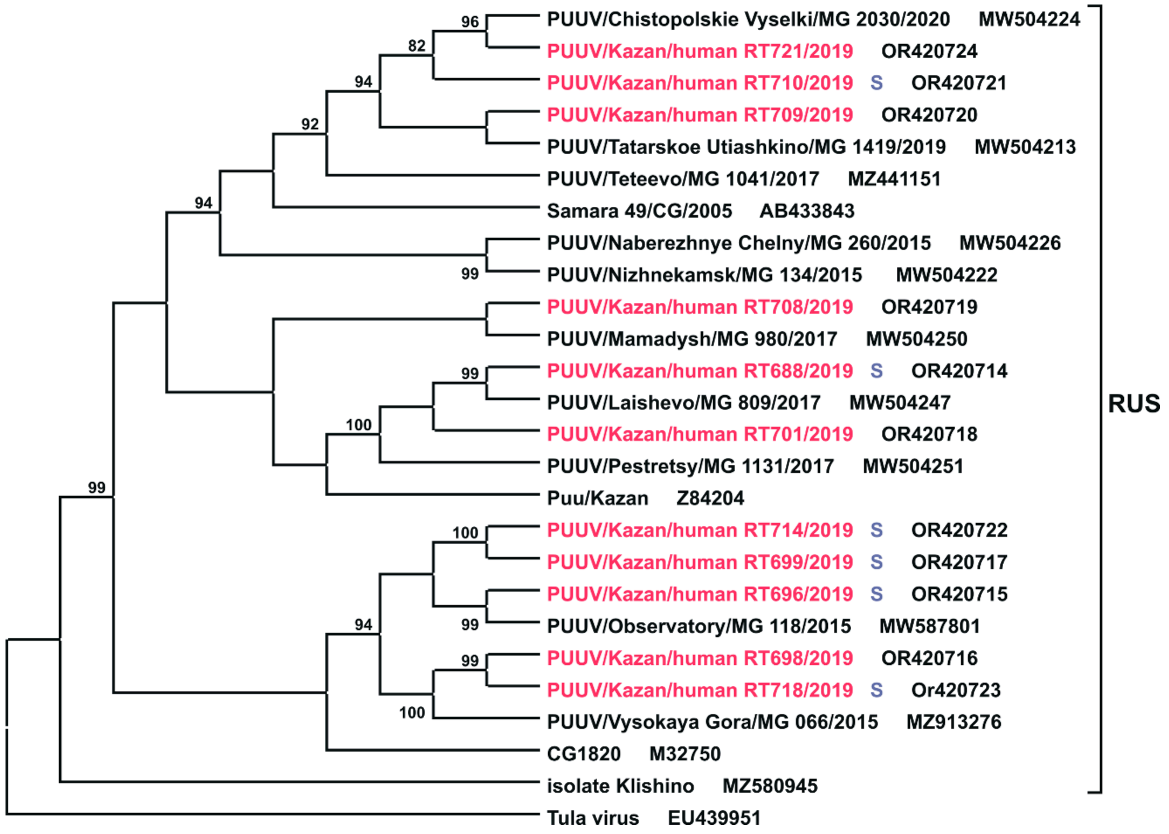

Figure S1. The phylogenetic analysis of PUUV from NE patients. Phylogenetic tree based on the S segment partial CDS nucleotide sequences (633 nt long, nt 43-1344, nucleotide sequence positions were numbered based on GenBank sequence Puu/Kazan strain, accession number Z84204) of the PUUV strains obtained from HFRS patients (shown in red) and reference strains from the bank voles. The maximum likelihood method was used for the analysis. The percentage of trees in which the associated taxa clustered together in the bootstrap test (1000 replicates) is shown next to the branches, and only values >70% are shown. The PUUV strains obtained from the ros-NE patients are marked by an "S".

Table S1. Analysis of kidney toxicity markers (ng/ml) in NE.

	NE	Ros-NE	control	Ros-Control	P value 1	2	3	4
**Albumin**	**824.6±869.8**	**431.8±857.8**	**1912.1±3266.2**	991.2±438.1	**0.039**	**0.020**	**0.028**	0.580
β2M	18.6±8.4	16.1±8.9	155.3±251.9	38.8±29.6	0.256	**0.001**	**0.002**	0.811
cystatin	471.2±1377.8	46.8±75.0	7.0±10.8	32.5±33.5	0.099	**<0.001**	0.609	0.112
NGAL	35.0±24.4	29.4±22.4	15.4±23.8	6.6±2.7	0.588	**0.001**	**0.041**	0.721
osteopontin	258.1±245.8	185.6±324.8	111.8±96.1	90.4±53.3	**0.014**	**0.021**	1.000	1.000
TFF3	149.2±78.8	131.5±36.4	146.3±229.3	81.6±60.4	0.287	0.060	0.204	0.422

1 – NE vs ros-NE; 2 – NE vs control; 3 – ros-NE vs ros-control; 4 – control vs ros-control.
